# Factors associated with vaccine intention in adults living in England who either did not want or had not yet decided to be vaccinated against COVID-19

**DOI:** 10.1080/21645515.2021.2002084

**Published:** 2021-12-17

**Authors:** Louis Goffe, Vivi Antonopoulou, Carly J Meyer, Fiona Graham, Mei Yee Tang, Jan Lecouturier, Aikaterini Grimani, Clare Bambra, Michael P. Kelly, Falko F Sniehotta

**Affiliations:** a NIHR Policy Research Unit in Behavioural Science – Population Health Sciences Institute, Faculty of Medical Sciences, Newcastle University, Newcastle upon Tyne, UK; b NIHR Policy Research Unit in Behavioural Science – Health Psychology Research Group, Department of Clinical, Education and Health Psychology, University College London, London, UK; c NIHR Policy Research Unit in Behavioural Science – Behavioural Science Group, Warwick Business School, University of Warwick, Coventry, UK; d NIHR Policy Research Unit in Behavioural Science – Primary Care Unit, East Forvie Building, Cambridge Biomedical Campus, Cambridge, UK; e Faculty of Behavioural, Management and Social Sciences - University of Twente, Enschede, The Netherlands

**Keywords:** COVID-19, vaccine, intention, hesitancy, attitudes, behavior, public health promotion

## Abstract

Early studies showed that 28–36% of UK adults were unsure or unwilling to be vaccinated against COVID-19. We wanted to identify which socio-demographic, socio-economic, personal health and psychological factors were associated with COVID-19 vaccine intentions (CVI) in adults living in England who did not want, yet to consider, or not sure whether to vaccinate. In October/November 2020, prior to vaccine availability, we surveyed adults stratified by gender, region, and deprivation, with additional purposive sampling of those aged 50 and over and those from an ethnic minority. Two hundred and ten did not want; 407 had yet to consider; and 1,043 were not sure whether to be vaccinated. Factors positively associated with CVI were: favorable vaccine views, trust in institutions associated with vaccine approval, vaccine subjective norms, anticipated regret of not having a vaccine, perceived vaccine benefits, perceived safety knowledge sufficiency, and a history of having an influenza vaccine. Factors negatively associated were: anti-lockdown views, and being a health or social care worker. Whilst showing significant relationships with CVI when analyzed in isolation, neighborhood deprivation and ethnicity did show an independent relationship to intention when all study measures were controlled for. Our findings suggest vaccine promotion focusing on the anticipated regret of not having a vaccine, the benefits of a mass COVID-19 immunization program, and the safety of a vaccine whilst ensuring or engendering trust in those bodies that brand a campaign may be most supportive of COVID-19 vaccine uptake.

## Introduction

As of 27 July 2021, the World Health Organization has recorded over 4.17 million COVID-19-related deaths.^1^ In England, there have been 131,170 deaths with COVID-19 on the death certificate.^2^ Around the world, governments have implemented numerous health protective measures to restrict the spread of the virus, including the widespread use of face coverings, social distancing measures, and lockdowns. Society has changed dramatically, particularly how we interact with others. Mass-inoculation to COVID-19 is seen as critical to control the pandemic and help return to usual social and economic ways of living.^3^ In April 2020, 1 month after the first national lockdown, the UK Government announced the formation of a Vaccine Taskforce to coordinate efforts to support research, production and delivery of a safe and effective vaccine against COVID-19.^4^ By November 2020, the Vaccine Taskforce had arranged the purchase of 357 million doses of seven different vaccines.^5^ On 8 January 2021, the Medicines and Healthcare products Regulatory Agency sanctioned a third COVID-19 vaccine for use across the UK.^6^

However, an efficacious and safe vaccine^7–9^ alone is not sufficient. A far-reaching immunization program that achieves high and equitable uptake will be required in order to deliver population-level immunological protection against COVID-19.^10^ A global study of 19 countries prior to the availability of a vaccine showed that acceptance ranged from 54.9% in Russia to 88.6% in China.^11^ Countries with communities that have historically opposed mass vaccination are likely to experience increased objections to a vaccine,^12^ with their views amplified through social media.^13^ The Centers for Disease Control and Prevention (CDC), the United States federal agency for public health protection, stated that dependent on the factors that impact on the rate of disease transmission, a minimum of 55% of the population will need to receive a vaccine, though this may be as high as 82%.^14^ As of publication, no UK Government or affiliated agency has published vaccination targets for population-level immunity.

Pandemics are experienced unequally, with higher rates of infection and death reported in those living in the most deprived communities.^15^ A systematic review and meta-analysis found that people of a lower socio-economic status suffered a significantly higher disease burden in both 1918 and 2009 influenza pandemics.^16^ This is no different for COVID-19. In countries where inequalities data has been reported, such as Spain,^17^ the US,^18^ Brazil,^19^ Chile,^20^ India,^21^ Sweden,^22^ and Germany^23^ it is consistently the poorest communities that have been the hardest hit. In England, whilst the most notable disparity in death rates is age, with those aged 80 or older at the greatest risk, the risk of dying was also higher in men, those living in more deprived areas, Black, Asian and Minority Ethnic groups, and those with comorbidities.^24^ There is also concern about the number of deaths among healthcare workers^25^ and those with obesity.^26^ As vaccines are seen as a critical tool in ending the global pandemic,^27^ a COVID-19 immunization strategy must therefore explicitly account for and seek to address these inequalities. This is to ensure that preventative measures are delivered to minimize health inequalities and support those most at risk from severe symptoms or death following a COVID-19 infection. The Government health departments of the four home nations of the UK are advised on COVID-19 vaccine safety, efficacy, and strategy by the Joint Committee on Vaccination and Immunization.^28^ It is an independent expert advisory panel with a diverse membership that publishes regular updates of their recommendations for COVID-19 vaccine priority groups that has informed the Government’s COVID-19 vaccine schedule.^29,30^

Research on UK populations prior to delivery of a national COVID-19 immunization program showed that a majority of respondents intended to have a COVID-19 vaccine.^31–34^ Two studies reported that 64% of respondents were ‘very likely’ to be vaccinated,^31,32^ and in a third 72% were ‘willing’ to be vaccinated.^34^ Intention to get vaccinated rose to 86% in a study with older adults and patients with chronic respiratory diseases.^33^ Such findings are positive, particularly given these figures are above the minimum population vaccine threshold of 55%.^14^ There is a small margin for error, given the discrepancy between the motivation to undertake a health protective action and the subsequent behavior,^35^ that intention assumes that there are no vaccine access issues, and that emergent more transmissible variants^36^ would likely increase the threshold required to achieve herd immunity.^14^ An increased focus is therefore required to better understand those who are either undecided, or who do not intend to have a COVID-19 vaccine.

Our aim was to identify those factors associated with COVID-19 vaccine intention (CVI) in adults living in England who did not want, had yet to consider, or were not sure whether to be vaccinated, specifically sampling those deemed either high priority for a vaccine or high risk of severe illness or death with COVID-19.

## Methods

We commissioned YouGov, a market research company, to deliver an online cross-sectional survey to their panel members living in England. YouGov were selected as they had access to a diverse panel of registered members that could deliver responses from our desired demographic groups across all regions of England.^37^ The survey ran from 23 October to 4 November 2020, which preceded the approval and availability of a COVID-19 vaccine in the UK and a second peak in the death rate across the country.^38^ As such, the survey captured behavioral intention and not COVID-19 vaccine behavior. We obtained ethical approval for the study from Newcastle University Research, Policy, Intelligence and Ethics Team (Reference: 4399/2020) on 18 September 2020.

### Policy, patient and public involvement

This work was developed in collaboration with policy makers at the National Infection Service at Public Health England.^39^ Patient and public involvement (PPI) was embedded throughout the research. The NIHR Policy Research Unit in Behavioural Science has its own dedicated PPI strategy group of seven external patient and public representatives. Two PPI members were involved in developing the proposal, all seven members contributed to the construction of the questionnaire, and five members reviewed the manuscript prior to submission.

### Sample

We recruited adults living in England who reported that they either did not want, were yet to consider, or not sure as to whether to be vaccinated against COVID-19. Due to the disproportionate impact of the disease on the elderly,^24^ the sampling frame specified within a target sample of 1,500 participants stratified by gender, geographical regions of England, and deprivation, and the inclusion of at least 500 respondents who were aged 50 or over, with at least 200 people aged 65 or over. Furthermore, due to associations between ethnicity and adverse outcomes from a COVID-19 infection,^24^ we specified a sample of at least 300 respondents who were from an ethnic minority. These sample targets were derived as a result of conversations with YouGov to ensure that the survey could be feasibly delivered within study time and cost constraints. YouGov uses a point-based program to incentivize survey participation. The points received are determined by survey length and are allocated to upon survey completion. Panel members accumulate points for completing surveys and are able to redeem these either for entries into prize draws or toward a cash payment.

### Questionnaire

#### Screening question

In order to achieve our desired focus to understand those who were either undecided, or who did not intend to have a COVID-19 vaccine, the survey started with a screening question to classify where respondents were in the decision-making process of having a COVID-19 vaccine. This was separate from the belief-based measure captured for CVI detailed below. This was developed applying the precaution adoption process model, a theory-based model that details the stages a person progresses through in deciding whether to act, or not, on a health protective behavior.^40^ It describes, in seven sequential stages, the process from being unaware of an issue to taking action to prevent ill health: stage 1, unaware of the issue; stage 2, unengaged by issue; stage 3, deciding about acting; stage 4, decided not to act; stage 5, decided to act; stage 6, acting; stage 7, maintenance. For our purpose we excluded stage 1, as we made the assumption that all participants were aware of the COVID-19 pandemic, and excluded stages 6 and 7, as a vaccine was not available at the time of data collection. The phrasing of the question was co-produced with members of the NIHR Policy Research Unit in Behavioural Science dedicated PPI strategy group. Respondents were asked: “Which of the following best describes your thoughts about getting vaccinated against coronavirus (COVID-19), once a vaccine is ready and is available to you?” where response options were: “I’ve not yet thought about getting vaccinated against coronavirus”; “I’m not yet sure about getting vaccinated against coronavirus, but will probably have a vaccine”; “I’m not yet sure about getting vaccinated against coronavirus, but will probably NOT have a vaccine”; “I’ve decided I don’t want to get vaccinated against coronavirus”; “I’ve decided I do want to get vaccinated against coronavirus.” Those who stated that they wanted to be vaccinated were asked no further questions. However, we were able to collect summary demographic information on both groups of respondents from YouGov’s panel profile information.

#### Study variables

##### Psychological measures

A number of theoretical models have been proposed in an attempt to identify the factors associated with health protective behavior. The Theory of Planned behavior (TPB) is the most widely used and influential social cognition model in predicting and explaining behavior.^41^ TPB proposes that behavior is determined by behavioral intention and perceived control. Intention is determined by attitudes toward the behavior (favorable or unfavorable), subjective norms (beliefs about whether significant others (e.g., family, friends) would approve of one performing the behavior in question) and perceived behavioral control (beliefs about one’s ability to perform or refrain from the behavior in various circumstances).

The Health Belief Model (HBM) has also been extensively applied to a wide range of, particularly preventive, health behaviors.^42^ HBM proposes that a health-related behavior depends on: perceived susceptibility, perceived severity, perceived benefits, perceived barriers, cues to action, self-efficacy and socio-demographic characteristics. It posits that health-related messages that target these factors will achieve optimal behavior change.^43^ While the HBM has a strong explanatory value, theoretical limitations due to undefined relationships between all constructs have been acknowledged.^44,45^

TPB has been criticized for excluding affective and unconscious influences on behavior.^46^ This has led some to propose further components, such as anticipated regret, vaccine knowledge, and past vaccine behavior^47,48^ to increase its predictive value. Anticipated regret is the concept where an individual realizes or imagines that the present situation could have been better if they had acted differently.^49,50^ Thus, Myers and Goodwin successfully applied an extended version of the TPB and incorporated additional constructs, including those from the HBM, to identify decisions that determine adults’ intention to vaccinate against pandemic swine flu.^48^

Given the level of overlap between these health behavior models (e.g., perceived barriers, perceived control, self-efficacy) and findings that support extension of the TPB, it is appropriate to combine model concepts along with specific COVID-19 factors, informed by studies published during the pandemic^31,51^ to predict COVID-19 vaccine intention. Therefore, we presented respondents with a series of statements based on these validated psychological measures to capture their beliefs on three COVID-19 areas: vaccine intention; pandemic beliefs; and beliefs about a potential vaccine. The available responses were distributed across a 5-point Likert scale with varying anchors. The details of these statements can be seen in Table 1 in which we detail the psychological theory that each item is based on and those studies that informed their development. This included a series of 12 questions relating to beliefs on COVID-19 pandemic misinformation and rumor shared on social media, informed by van Mulukom et al.’s review on the antecedents and consequences of COVID-19 conspiracy beliefs.^51^ As it had been found that there was a negative association between COVID-19 conspiracy beliefs and COVID-19 health protective behaviors, such views may also influence CVI.^52^

**Table 1.** Details of the psychological measure captured, the supporting theory, and those studies that informed their development

COVID-19 belief area	Factor	Psychological theory	Statements used to capture	Informing study	Responses
COVID-19 vaccine intention	COVID-19 vaccine intention	Theory of planned behavior	When it’s available to me, I will have a coronavirus vaccine	Sherman et al.^31^	Strongly disagree Disagree Neither agree nor disagree Agree Strongly agree
Beliefs regarding the COVID-19 pandemic	Misinformation and rumor on social media	NA	Coronavirus is no worse than seasonal flu Social distancing has done more harm than good The wearing of face coverings in indoor public spaces is unnecessary Lockdown measures are pointless and are damaging the economy Lockdown measures are a violation of my basic rights and freedoms Coronavirus probably came from a laboratory Washing hands regularly is essential to protect each other from coronavirus The symptoms that most people blame on coronavirus appear to be linked to 5 G network radiation There is no hard evidence that coronavirus really exists The number of people reported as dying from coronavirus is being deliberately exaggerated by the authorities The current pandemic is part of a global effort to force everyone to be vaccinated to benefit the vaccine companies Mass coronavirus vaccination is a ploy by environmental lobbyists to sterilize billions of people to reduce population growth	van Mulukom et al.^51^	Strongly disagree Disagree Neither agree nor disagree Agree Strongly agree
Beliefs regarding the COVID-19 pandemic	Perceived severity of a COVID-19 infection	Health belief model	Complications from coronavirus would be serious for me I will be very sick if I get coronavirus	Myers & Goodwin^48^	Strongly disagree Disagree Neither agree nor disagree Agree Strongly agree
Beliefs regarding the COVID-19 pandemic	Perceived susceptibility to COVID-19 infection	Health belief model	I believe that I’m at high risk of catching coronavirus compared to others	Myers & Goodwin^48^	Strongly disagree Disagree Neither agree nor disagree Agree Strongly agree
Beliefs regarding the COVID-19 pandemic	Trust in the NHS and the UK Government body approving a COVID-19 vaccine	NA	I believe that a coronavirus vaccine approved by a UK Government body, will be very safe I believe that a coronavirus vaccine approved by the NHS, will be very safe	Sherman et al.^31^	Strongly disagree Disagree Neither agree nor disagree Agree Strongly agree
Beliefs regarding a potential COVID-19 vaccine	COVID-19 vaccine attitudes	Theory of planned behavior	I feel that having a vaccine against coronavirus this year would be:	Myers & Goodwin^48^	Worthless to Valuable Harmful to Beneficial Painful to Tolerable
Beliefs regarding a potential COVID-19 vaccine	COVID-19 vaccine subjective norms	Theory of planned behavior	My family would expect me to be vaccinated for coronavirus My GP would expect me to be vaccinated for coronavirus I believe that a coronavirus vaccine approved by the NHS, will be very safe	Myers & Goodwin^48^	Strongly disagree Disagree Neither agree nor disagree Agree Strongly agree
Beliefs regarding a potential COVID-19 vaccine	COVID-19 vaccine perceived control	Theory of planned behavior	I feel in total control as to whether I will have a coronavirus vaccine	Myers & Goodwin^48^	Strongly disagree Disagree Neither agree nor disagree Agree Strongly agree
Beliefs regarding a potential COVID-19 vaccine	COVID-19 vaccine anticipated regret	NA	Imagine that you caught coronavirus, but that a vaccine might have prevented it Imagine that you caught coronavirus and passed on to a friend, but that a vaccine might have prevented it Imagine that you caught coronavirus and passed on to a family member, but that a vaccine might have prevented it	Myers & Goodwin^48^ Ziarnowski et al.^50^	Strongly disagree Disagree Neither agree nor disagree Agree Strongly agree
Beliefs regarding a potential COVID-19 vaccine	COVID-19 vaccine perceived safety knowledge sufficiency	NA	I know enough about the safety of a coronavirus vaccine to make an informed decision about whether or not to get vaccinated for coronavirus	Sherman et al.^31^	Strongly disagree Disagree Neither agree nor disagree Agree Strongly agree
Beliefs regarding a potential COVID-19 vaccine	Perceived benefits a mass COVID-19 immunization programme	Health belief model	If I have a coronavirus vaccine, I’m confident that I will not be able to catch the coronavirus If I have a coronavirus vaccine, and still caught coronavirus, the severity of my illness will be reduced If I have a coronavirus vaccine, I won’t be able to spread coronavirus to others If I have a coronavirus vaccine, I won’t have to socially distance to protect others from coronavirus Mass coronavirus vaccination, will protect the NHS Mass coronavirus vaccination, will help the country get back to normal	Myers & Goodwin^48^ Sherman et al.^31^	Strongly disagree Disagree Neither agree nor disagree Agree Strongly agree

##### Socio-demographic and socio-economic measures

YouGov’s panel profile information provided the following demographic information: age; gender; ethnicity; and the Indices of Multiple Deprivation decile (IMD), a measure of neighborhood deprivation based on the respondent’s home postcode that accounts for economic, social, and physical environmental factors.^53^ We also asked respondents if they considered themselves to be a key worker in one of the industries as defined by the Office of National Statistics.^54^ Each of these variables were associated with a disparity in outcome from COVID-19 by Public Health England,^24^ as well as informing the JCVI advice on priority groups for COVID-19 vaccination.^30^

##### Personal health

We captured respondents’ Body Mass Index (BMI) by asking for their self-reported height and weight measurements. We asked them about their general health using the same question and response options as used in The Health Survey for England which ranged from “Very good” to “Very bad.”^55^ We asked them if they have previously had a COVID-19 infection and whether they have been shielding (remaining at home and minimizing face-to-face contact to avoid infection) at any time from COVID-19 during the pandemic. These were important to capture as JCVI had specifically specified that underlying health conditions, inclusive of obesity, may result in a higher risk of serious disease and mortality.^30^ We also captured respondents’ seasonal influenza vaccine history, as this is a past related behavior, an item of the extended Theory of Planned Behavior.^48^

### Analysis

We used hierarchical linear regression (HLR) to identify those factors associated with CVI, the outcome variable of interest. Prior to construction, to reduce the dimensions of the response data from the ‘misinformation and rumour shared on social media’ items and simplify interpretation, we ran a principal component analysis with varimax rotation. To check the reliability within each theoretical item, we calculated Cronbach’s alpha for those that were constructed of three or more responses and Pearson correlation coefficient for those that only consisted of two responses. We generated a correlation matrix using all HLR variables to examine for any bivariate associations. The HLR consisted of four blocks of variables that were selected a priori based on their theoretical and logical relevance and not through stepwise statistical methods. Where appropriate certain variables were converted to dummy variables. Block 1 consisted of socio-demographic and socio-economic variables; Block 2 consisted of personal health variables; Block 3 consisted of variables capturing beliefs regarding the COVID-19 pandemic; and Block 4 consisted of variables capturing beliefs regarding a potential COVID-19 vaccine. The fit of each sequential regression was measured by multiple and adjusted R2. We applied the variance inflation factor as a diagnostic to identify any possible multicollinearity within HLR.^56^ Data analysis was carried out using R.^57^

## Results

A total of 3,039 people completed the screening question about getting vaccinated against COVID-19 where: 407 (13.4%) had not yet thought about getting vaccinated; 783 (25.8%) were not yet sure, but will probably have a vaccine; 260 (8.6%) were not yet sure, but will probably not have a vaccine; 210 (6.9%) had decided they did not want to get vaccinated; and 1,379 (45.4%) had decided they did want to get vaccinated against COVID-19. This resulted in 1,660 respondents that completed the full questionnaire. With regard to risk factors and high priority groups, 714 (43.0%) were male, 538 (32.4%) were aged 50 or over, 363 (21.9%) were from an ethnic minority, 453 (27.3%) lived in the three most deprived IMD deciles, 320 (19.3%) had been shielding from COVID-19, and 283 (17.0%) were obese. Given the high prevalence of each risk factor and likely comorbidity, there was a substantial proportion of respondents who had two or more risk factors. A detailed summary of respondent characteristics can be seen in Table 2. The mean and standard deviation of the responses for each of the belief-based statements as well as the Cronbach’s alpha or Pearson correlation coefficient where appropriate for each measure can be seen in Table 3.

**Table 2.** Summary statistics of respondent’s socio-demographic and health characteristics

Characteristic	Level	n
Gender	Female	946 (57.0%)
	Male	714 (43.0%)
Age	Under 50	1122 (67.6%)
	50–64	332 (20.0%)
	65 and over	206 (12.4%)
Ethnicity	White	1297 (78.1%)
	English/Welsh/Scottish/Northern Irish/British	1209 (72.8%)
	Irish	17 (1.0%)
	Any other White background	71 (4.3%)
	Mixed/multiple ethnic groups	83 (5.0%)
	White and Black Caribbean	20 (1.2%)
	White and Black African	7 (0.4%)
	White and Asian	31 (1.9%)
	Any other Mixed/Multiple ethnic background	25 (1.5%)
	Asian/Asian British	180 (10.8%)
	Indian	69 (4.2%)
	Pakistani	36 (2.2%)
	Bangladeshi	25 (1.5%)
	Chinese	26 (1.6%)
	Any other Asian background	24 (1.4%)
	Black/African/Caribbean/Black British	88 (5.3%)
	African	45 (2.7%)
	Caribbean	31 (1.9%)
	Any other Black/African/Caribbean background	12 (0.7%)
	Other ethnic group	12 (0.7%)
	Arab	3 (0.2%)
	Any other ethnic group	9 (0.5%)
England region	London	286 (17.2%)
	East Midlands	141 (8.5%)
	East of England	155 (9.3%)
	North East	67 (4.0%)
	North West	198 (11.9%)
	South East	281 (16.9%)
	South West	163 (9.8%)
	West Midlands	179 (10.8%)
	Yorkshire and the Humber	190 (11.4%)
Deciles of Indices of Multiple Deprivation	1 (most deprived)	137 (8.3%)
	2	170 (10.2%)
	3	146 (8.8%)
	4	157 (9.5%)
	5	166 (10.0%)
	6	145 (8.7%)
	7	172 (10.4%)
	8	173 (10.4%)
	9	187 (11.3%)
	10	204 (12.3%)
	Missing	3 (0.2%)
Key worker	Not a key worker	585 (35.2%)
	Not sure	103 (6.2%)
	Key worker – Health and social care	110 (6.6%)
	Key worker – Education and childcare	129 (7.8%)
	Key worker – Utilities and communication	28 (1.7%)
	Key worker – Food and necessary goods	70 (4.2%)
	Key worker – Transport	37 (2.2%)
	Key worker – Key public services	48 (2.9%)
	Key worker – Public safety and national security	15 (0.9%)
	Key worker – National and local governments	34 (2.0%)
	Not in work	501 (30.2%)
BMI	Underweight	67 (4.0%)
	Healthy weight	646 (38.9%)
	Overweight	469 (28.3%)
	Obese	283 (17.0%)
	Missing	195 (11.7%)
Previously had COVID-19	Not had COVID-19	1336 (80.5%)
	Had COVID-19	284 (17.1%)
	Missing	40 (2.4%)
Shielding from COVID-19	Yes	320 (19.3%)
	No	1340 (80.7%)
General health	Very bad	13 (0.8%)
	Bad	60 (3.6%)
	Fair	390 (23.5%)
	Good	773 (46.6%)
	Very good	358 (21.6%)
	Missing	66 (4.0%)
Seasonal influenza vaccine frequency	Never	794 (47.8%)
	Rarely	275 (16.6%)
	Some years	145 (8.7%)
	Most years	140 (8.4%)
	Every year	306 (18.4%)

**Table 3.** The psychological measures and the summary response for each statement and either the Cronbach’s alpha or Pearson correlation coefficient for each measure where appropriate

Measurement	Statements used to capture measure	Response: 5-point Likert scale	Mean (SD)	Cronbach’s alpha/Pearson correlation
COVID-19 vaccine intention	When it’s available to me, I will have a coronavirus vaccine.	Strongly disagree (1) – Strongly agree (5)	3.11 (1.07)	-
Misinformation and rumor on social media	Coronavirus is no worse than seasonal flu.	Strongly disagree (1) – Strongly agree (5)	2.19 (1.14)	0.90
	Social distancing has done more harm than good.		2.31 (1.18)	
	The wearing of face coverings in indoor public spaces is unnecessary.		2.09 (1.17)	
	Lockdown measures are pointless and are damaging the economy.		2.60 (1.26)	
	Lockdown measures are a violation of my basic rights and freedoms.		2.42 (1.27)	
	Coronavirus probably came from a laboratory.		3.14 (1.21)	
	Washing hands regularly is essential to protect each other from coronavirus.		4.34 (0.84)	
	The symptoms that most people blame on coronavirus appear to be linked to 5 G network radiation.		1.58 (0.89)	
	There is no hard evidence that coronavirus really exists.		1.70 (0.97)	
	The number of people reported as dying from coronavirus is being deliberately exaggerated by the authorities.		2.51 (1.30)	
	The current pandemic is part of a global effort to force everyone to be vaccinated to benefit the vaccine companies.		2.00 (1.12)	
	Mass coronavirus vaccination is a ploy by environmental lobbyists to sterilize billions of people to reduce population growth.		1.84 (1.05)	
Perceived severity of a COVID-19 infection	Complications from coronavirus would be serious for me.	Strongly disagree (1) – Strongly agree (5)	2.98 (1.11)	0.81
	I will be very sick if I get coronavirus.		3.03 (1.05)	
Perceived susceptibility to COVID-19 infection	I believe that I’m at high risk of catching coronavirus compared to others.	Strongly disagree (1) – Strongly agree (5)	2.55 (1.11)	-
Trust in the NHS and the UK Government body approving a COVID-19 vaccine	I believe that a coronavirus vaccine approved by a UK Government body, will be very safe.	Strongly disagree (1) – Strongly agree (5)	2.96 (1.04)	0.71
	I believe that a coronavirus vaccine approved by the NHS, will be very safe.		3.36 (1.07)	
COVID-19 vaccine attitudes	I feel that having a vaccine against coronavirus this year would be:	Worthless (1) – Valuable (5)	3.71 (1.25)	0.90
		Harmful (1) – Beneficial (5)	3.73 (1.23)	
		Painful (1) – Tolerable (5)	3.84 (1.18)	
COVID-19 vaccine subjective norms	My family would expect me to be vaccinated for coronavirus.	Strongly disagree (1) – Strongly agree (5)	3.08 (1.11)	0.74
	My GP would expect me to be vaccinated for coronavirus.		3.35 (1.03)	
	I will feel under social pressure to be vaccinated for coronavirus.		3.06 (1.16)	
COVID-19 vaccine perceived control	I feel in total control as to whether I will have a coronavirus vaccine.	Strongly disagree (1) – Strongly agree (5)	3.45 (1.11)	-
COVID-19 vaccine anticipated regret	Imagine that you caught coronavirus, but that a vaccine might have prevented it.	Not at all (1) – A great deal (5)	3.31 (1.36)	0.95
	Imagine that you caught coronavirus and passed on to a friend, but that a vaccine might have prevented it.		3.61 (1.36)	
	Imagine that you caught coronavirus and passed on to a family member, but that a vaccine might have prevented it.		3.72 (1.38)	
Perceived safety knowledge sufficiency	I know enough about the safety of a coronavirus vaccine to make an informed decision about whether or not to get vaccinated for coronavirus.	Strongly disagree (1) – Strongly agree (5)	2.56 (1.11)	-
Perceived benefits a mass COVID-19 immunization programme	If I have a coronavirus vaccine, I’m confident that I will not be able to catch the coronavirus.	Strongly disagree (1) – Strongly agree (5)	2.62 (0.95)	0.85
	If I have a coronavirus vaccine, and still caught coronavirus, the severity of my illness will be reduced.		3.10 (0.92)	
	If I have a coronavirus vaccine, I won’t be able to spread coronavirus to others.		2.55 (0.94)	
	If I have a coronavirus vaccine, I won’t have to socially distance to protect others from coronavirus.		2.42 (0.99)	
	Mass coronavirus vaccination, will protect the NHS.		3.46 (1.08)	
	Mass coronavirus vaccination, will help the country get back to normal.		3.40 (1.11)	

### Principal component analysis with varimax rotation

To measure sampling adequacy of the responses to misinformation and rumor shared on social media we used Kaiser–Meyer–Olkin measure of sampling adequacy, which was very high (0.928), and Bartlett’s Test of Sphericity, which was highly significant (χ^2^(66) = 10,284.73, *p* < .001). Two factors resulted from the principal component analysis with varimax rotation. The loading scores for these factors can be seen in Table 4. We interpreted the first factor as relating to those that hold views that are in opposition to the lockdown measures imposed by the UK Government. The six statements with the highest loading scores were: “Lockdown measures are pointless and are damaging the economy”; “Lockdown measures are a violation of my basic rights and freedoms”; “Social distancing has done more harm than good”; “The wearing of face coverings in indoor public spaces is unnecessary”; “Coronavirus is no worse than seasonal flu”; and “The number of people reported as dying from coronavirus is being deliberately exaggerated by the authorities.” We interpreted the second factor as relating to those respondents that held conspiratorial views regarding the pandemic. The highest loading scores were on these four statements: “Mass coronavirus vaccination is a ploy by environmental lobbyists to sterilise billions of people to reduce population growth”; “The symptoms that most people blame on coronavirus appear to be linked to 5 G network radiation”; “The current pandemic is part of a global effort to force everyone to be vaccinated to benefit the vaccine companies”; “There is no hard evidence that coronavirus really exists; Coronavirus probably came from a laboratory.”

**Table 4.** The loading scores resulting from the principal component analysis with varimax rotation of the responses to the 12 belief-based statements on misinformation and rumors shared on social media

Statement	Anti-lockdown proponent	Conspiracy theorists
Coronavirus is no worse than seasonal flu.	0.679	0.352
Social distancing has done more harm than good.	0.776	0.243
The wearing of face coverings in indoor public spaces is unnecessary.	0.757	0.235
Lockdown measures are pointless and are damaging the economy.	0.864	0.157
Lockdown measures are a violation of my basic rights and freedoms.	0.825	0.234
Coronavirus probably came from a laboratory.	0.223	0.505
Washing hands regularly is essential to protect each other from coronavirus.	−0.297	−0.326
The symptoms that most people blame on coronavirus appear to be linked to 5 G network radiation.	0.067	0.807
There is no hard evidence that coronavirus really exists.	0.360	0.701
The number of people reported as dying from coronavirus is being deliberately exaggerated by the authorities.	0.648	0.476
The current pandemic is part of a global effort to force everyone to be vaccinated to benefit the vaccine companies.	0.349	0.772
Mass coronavirus vaccination is a ploy by environmental lobbyists to sterilize billions of people to reduce population growth.	0.253	0.836

### COVID-19 vaccine intention

**Figure 1.**
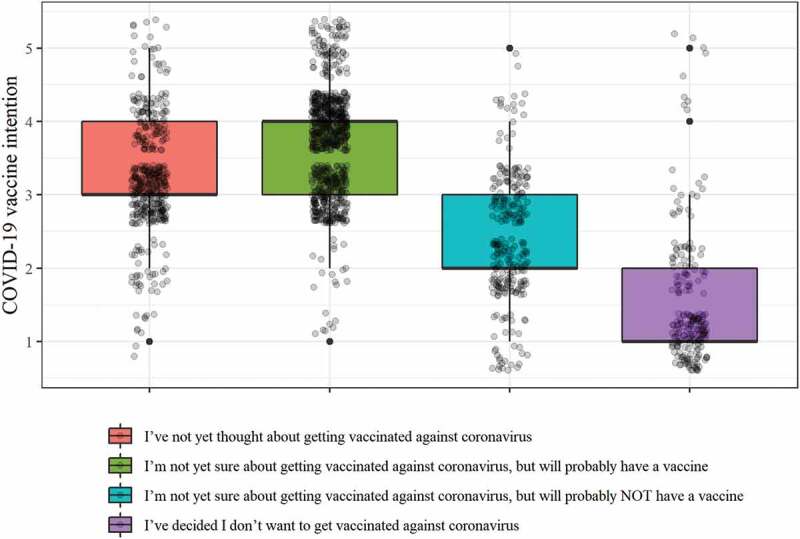
Distribution of CVI response grouped according to where respondents were in the decision-making process regarding having a COVID-19 vaccine.

The distribution of respondents’ CVI grouped by their response to the screening question can be seen in Figure 1. CVI was highest in those who responded that they were not yet sure about getting vaccinated against COVID-19, but will probably have a vaccine (M = 3.70, SD = 0.71); next was those who had not yet thought about getting a vaccine (M = 3.23, SD = 0.78); followed by those who were not yet sure about getting vaccinated against coronavirus, but will probably not have a vaccine (M = 2.41, SD = 0.82); with those that had responded that they had decided not to have a vaccine with the lowest average CVI (M = 1.53, SD = 0.89).

A summary from the hierarchical linear regression can be seen in Table 5. The variance inflation factor scores are reported for the Block 4 regression, with all factors scoring under 2.5, indicating low collinearity. The complete summary of each regression block can be seen in Tables S1 to S4 in Supplementary materials. When including all variables detailed above in the analysis, they explained 60% of the variation in our study population’s responses to CVI. Factors that had a significant positive association with CVI were as follows: COVID-19 vaccine attitudes (β = 0.227, *p* < .001), trust in the NHS and the UK Government body approving a COVID-19 vaccine (β = 0.205, *p* < .001), COVID-19 vaccine subjective norms (β = 0.193, *p* < .001), COVID-19 vaccine anticipated regret (β = 0.170, *p* < .001), perceived benefits of a mass COVID-19 immunization program (β = 0.149, *p* < .001), perceived safety knowledge sufficiency (β = 0.050, *p* < .01), and historical seasonal influenza vaccine behavior (β = 0.043, *p* < .05). Factors that had a significant negative association were: being an anti-lockdown proponent (β = −0.051, *p* < .05), and being a health or social care worker (β = −0.036, *p* < .05). Beliefs on the perceived severity of a COVID-19 infection and holding pandemic conspiratorial views were significantly associated with CVI when analyzed alongside demographics and personal health variables. Though, their ability to predict CVI was no longer significant once views on a COVID-19 vaccine were accounted for.

**Table 5.** The summary results for the four hierarchical linear regressions, reporting the β scores (standardized coefficient) for each predictor with * used to indicate significance, as well as the variance inflation factor scores for the Block 4 regression. The adjusted R^2^ details the variance in response to CVI accounted for by each block

Factor	Block 1 β	Block 2 β	Block 3 β	Block 4 β	Variance inflation factor (Block 4)
Age	0.044	−0.035	−0.025	0.004	1.352
Gender: Female	−0.015	−0.040	−0.024	−0.018	1.094
Ethnicity: Black and mixed Black	−0.053*	−0.018	−0.007	0.020	1.086
IMD	0.067**	0.072**	0.028	0.032	1.069
Is a health & social care key worker	−0.020	−0.044	−0.031	−0.036*	1.054
BMI		0.043	0.026	0.004	1.115
Previously had COVID-19		−0.089***	−0.027	−0.002	1.053
Have been shielding from COVID-19		−0.019	−0.030	−0.007	1.172
General health		−0.025	−0.012	−0.028	1.372
Flu vaccine history: increasing regularity		0.258***	0.132***	0.043*	1.375
Anti-lockdown proponent			−0.168***	−0.051*	1.496
Conspiracy theorist			−0.109***	−0.001	1.438
Perceived severity of a COVID-19 infection			0.074**	0.007	2.089
Perceived susceptibility to COVID-19 infection			0.003	−0.027	1.710
Trust in the NHS and the UK Government body approving a COVID-19 vaccine			0.500***	0.205***	2.231
COVID-19 vaccine attitude				0.227***	2.328
COVID-19 vaccine subjective norms				0.193***	1.468
COVID-19 vaccine perceived control				0.010	1.232
COVID-19 vaccine anticipated regret				0.170***	2.174
Perceived safety knowledge sufficiency				0.050**	1.130
Perceived benefits of COVID-19 immunization				0.149***	1.923
Adjusted R^2^	0.009	0.078	0.452	0.595	
Adjusted R^2^ change		0.069	0.374	0.143	

* p < .05; ** p < .01; *** p < .001.

When assessed in isolation (see Table 5 Block 1), socio-demographic and socio-economic factors only explained a small percentage (1%) of the variance in our study population’s responses that predict CVI. Neither age nor gender was found to be significantly associated with CVI. We assessed all ethnic groups within our study population, where the only ethnic group that had a significant relationship with CVI were of either Black or mixed-Black heritage. They were associated with lower CVI. Additionally, living in an increasingly deprived area was also associated with lower CVI. Once beliefs on the pandemic and vaccines were accounted for, these relationships were no longer significant. However, those working in either health or social care remained significant. Respondents working in this sector were associated with lower CVI. This may be a result of the higher rate of COVID-19 infection (23%) compared to the rest of our study respondents (17%).

Our analysis of CVI in those at high-risk to the virus because of an underlying health problem found no significant association with BMI, whether a respondent had been shielding during the pandemic, or their general health. This corresponds to our finding that neither severity nor susceptibility to COVID-19 were significant predictors. Those who regularly have the influenza vaccination were more likely to have increased CVI.

## Discussion

### Principal findings

While only a minority (6.9%) had decided against having a COVID-19 vaccine, a critical mass of 47.7% had yet to make a conclusive decision regarding whether they would have a COVID-19 vaccine. If CVI is a reliable measure of COVID-19 vaccine uptake, then a concerted effort would be required to ensure that a sufficient proportion of the population of England is immunized against the virus. Irrespective of access issues, they will need convincing to have a vaccine against COVID-19, if we are to achieve the substantial levels of COVID-19 vaccine uptake required to provide England with population-level immunity.

To ensure equity of the delivery of the COVID-19 immunization program, particular consideration will have to be given to those who live in more deprived areas, and to those who are of either Black or mixed Black heritage. Ease of access to a vaccine, along with a comprehensive and targeted messaging program will be critical to reduce the health inequalities that have been observed as a result of the pandemic.^15^

Our findings suggest that in addition to having a positive attitude toward a COVID-19 vaccine, CVI will increase as one perceives a favorable view of COVID-19 vaccination from those close to us. In addition to these established factors from the TPB, we also identified that greater trust in vaccine authorizing bodies, anticipated regret of not being vaccinated, a favorable perception of the benefits of a mass-immunization program, and improving safety knowledge are related to higher CVI. Our research also highlights the potential challenges from those holding anti-lockdown views, but also from those who are health and social care workers. However, it must be noted that health and social care workers only comprised a small number within our study population (110 (6.6%) of our respondents) and therefore this result should be treated with caution (as these views may not be representative of the wider population of professionals).

### Strengths and limitations

We delivered a large-scale survey that captured and assessed CVI within the high priority and high-risk socio-demographic groups specified by the independent expert committee that advises UK health departments on COVID-19 immunization policy.

The limitations of our study were that we only used online data collection methods. Given the urgency of our findings to our policy partner, this was the only method deemed feasible. While most households in Great Britain have internet access, it is not universal.^58^ We will have therefore not captured the beliefs of some of the most vulnerable in society, such as rough sleepers, who have limited internet access.^59^ As we may not have captured the beliefs of those most vulnerable in our society and those who were screened out of our survey, it is possible that the relationship we observed in relation to deprivation (IMD quintiles) may be further exacerbated.^32^ The cross-sectional design of our survey means that we cannot infer causality. The lack of temporal data also means that the findings were reflective of the specific time point at which they were collected. This was prior to the approval for use of any COVID-19 vaccine and the subsequent reporting. It is unknown how the increase of COVID-19 vaccine information across traditional and social media impacted on those factors collected as part of this study. Furthermore, the statistical method that we applied infers a direct relationship between predictors and CVI. Alternative methods that consider predictors as a network of constructs may be preferable, such as structural equation modeling.^60^ We captured and assessed intention and not COVID-19 vaccine behavior. Currently, the relationship between COVID-19 vaccine intention and the behavior is unknown, prior research suggests that intention overestimates actual behavior.^61^

### Comparisons with other studies

While we did not structure our study to achieve a population-representative sample, we did achieve a good spread of respondents across a range of socio-demographic factors, see Table 2. Overall, 71.1% of our respondents stated that they either would or probably would have a vaccine against COVID-19. This is markedly lower than those that responded positively to having a COVID-19 vaccine in China (88.6%), but comparable to the European nations of Spain (74.5%), Italy (68.7%), and Germany (68.4%) and notably higher than both France (58.9%) and Poland (56.3%).^11^ In the UK, our findings are similar to Freeman et al. whose study found that 71.7% of UK adults were willing to be vaccinated,^34^ and higher than both Sherman et al. and Paul et al. studies of UK adults, who reported 64% and 63.5%, respectively, as being very likely to vaccinate against COVID-19.^31,32^ Our figure is lower than that of 86% obtained by Williams et al.,^33^ however, they exclusively sampled people identified as being at high risk from the disease. This high level of intention across studies is reflected in the media reporting of the early stages of a vaccine rollout in the UK, with example case studies of demand surpassing supply.^62^ Despite this, and the fact that only 6.9% of our study’s respondents, 11.7% in Freeman et al.,^34^ 9% in Sherman et al.,^31^ and 14.0% in Paul et al.,^32^ reported that they do not intend to have a vaccine, there remains a substantial proportion identified in all these studies who were undecided prior to the approval and subsequent reporting of effective and safe COVID-19 vaccines.

Globally, trust in respective governments was positively associated with increased COVID-19 vaccine acceptance, which was reflected in our related finding of “trust in the NHS and the UK Government body approving a COVID-19 vaccine.”^11^ Within the UK, similar to our findings, Sherman et al. found that it was COVID-19 vaccination beliefs and attitudes that accounted for the greatest proportion of variation in CVI.^31^ They also identified that perceived risk to others (but not oneself) was associated with CVI, complementing our findings that perceived susceptibility is not associated with CVI though protecting the NHS and the wider benefits of a mass immunization program are predictors of CVI. Freeman et al. identified the collective importance (community rather than individual considerations), efficacy, side-effects, speed of vaccine development, excessive mistrust (this included holding conspiratorial beliefs), and positive healthcare experiences were associated with CVI.^34^ In line with our findings, Freeman et al. suggested that the benefits of mass immunization should be the focus of public health messaging, particularly those which are prosocial.^34^ Comparatively, Paul et al. suggested that addressing the mistrust of vaccine benefits and the potential side effects should be the focus of public health messaging in support of a COVID-19 immunization campaign.^32^

With regard to socio-demographics, all aforementioned studies identified ethnicity as a critical factor when considering CVI.^31,32,34^ As in our findings, the concern regarding lower levels of CVI in those of Black as well as mixed ethnic heritage was highlighted.^34^ Additionally, both Paul et al. and Freeman et al. found a negative association with deprivation and CVI,^32,34^ which supports our findings that COVID-19 vaccine uptake may be lower in more deprived areas. Although Sherman et al. and Freeman et al. found an association with age,^31,34^ where younger adults had lower CVI, though this was not observed in either our or Paul et al.’s study.^32^

### Implications

The aim and scope of our research was developed in collaboration with policy makers within the national infection service at Public Health England.^39^ It was constructed to specifically inform and support policy development with regard to COVID-19 immunization strategy in England. Our findings suggest that public health messaging that supports uptake of a COVID-19 vaccine should focus on: the regret of not being vaccinated; the benefits of a mass-immunization program, and improving the public’s knowledge regarding the safety of the approved COVID-19 vaccines. To address inequalities in terms of ethnicity and also neighborhood level deprivation, targeted efforts are needed to support the implementation of a COVID-19 immunization strategy.

Trust in those bodies associated with vaccine approval and delivery will also be crucial. For example, the UK Government’s decision to abolish Public Health England in August 2020 during the pandemic may have a negative impact as they are one of the key agencies identified in developing the UK COVID-19 vaccine delivery plan.^10^ Messages that use individual susceptibility and severity to COVID-19 are unlikely to be as effective. Conspiratorial views were associated with lower CVI but are unlikely to significantly impact on population-level immunization when the issues detail above are addressed. Additionally, once an individual has accepted a vaccine conspiracy theory, it is challenging to convince them of its falsehood.^63^ It is therefore more effective to implement preventative methods that can combat the spread of false information.^64^ Those holding anti-lockdown views, will likely be challenging to vaccinate, despite accounting for those factors associated with increased CVI. As such sentiment is a novel construct that has emerged from the pandemic, it is unknown how deep such views are held.

Policy makers must ensure that public health messaging and access to vaccine reaches those groups of concern identified here. Those who are of Black or mixed Black heritage and those who live in more deprived areas were associated with lower CVI. Therefore, they must be specifically targeted for support to ensure that the disparities that have been observed with regard to adverse outcomes from a COVID-19 infection^15,24^ are not further widened. Respondents who have previously had COVID-19 are more likely to be difficult to encourage to be vaccinated. This may be due to a belief that they would have some form of antibody protection, and that any subsequent COVID-19 infection would be mild.^65^ Our findings also suggest that there may be resistance in some health and social care workers to having a COVID-19 vaccine. Stead et al. provided a series of recommendations to improve uptake of seasonal influenza vaccine by healthcare worker, which could be adopted by policy makers. These included employing multiple-communication strategies, provision of incentives, such as giveaways and free food during vaccination, and management support.^66^ Innovative methods delivering vaccination education through online games has also shown promise in increasing uptake of influenza vaccination in student nurses.^67^

The significance of COVID-19 subjective norms suggests that it would be beneficial, where possible to enhance the view that the people we both care about and respect would want us to be vaccinated. Wood and Schulman proposed the idea of ‘increased observability,’ making the act of having been vaccinated visible, referencing the Livestrong bracelets and the Apple iPod headphones, where people become walking advertisements as well digital badges that could be displayed on one’s social media profile.^38^ Such measures will need to be carefully balanced and tested to ensure that they do not lead to stigmatizing of those who have not been vaccinated.

### Future work

The grouping of CVI by the stage in which respondents were in the decision-making process warrants further analysis. The application of the precaution adoption process model provides a suitable theory to identify differences between groups and what factors might support increased CVI dependent on whether a person has made the choice to have a COVID-19 vaccine or not. At the time of submission, the relationship between intention and COVID-19 vaccine behavior is unknown, in addition to how the emergence and reporting of new COVID-19 variants might impact both vaccine intention and uptake.^68^ We have proposed what we consider should form the components of public health messaging promoting a COVID-19 vaccine and which groups in our society should have greater care and attention regarding delivery, this could complement the findings from a rapid systematic review of public responses to vaccine promotional messaging during previous pandemics.^69^ Future research could examine the effectiveness of such public health communication strategies. Moreover, discrete choice experiments could explore the effectiveness of different messages within different target demographic groups. It has been suggested that more should be done to increase and improve engagement with those who have concerns regarding vaccines,^63^ novel methods beyond traditional mass-messaging could play a role. Methods such as online games^67^ and chatbots^70^ have shown encouraging results, but are yet unproven at the population level.

## Conclusion

COVID-19 vaccine intention in England prior to the delivery of a population-level immunization program is socio-economically and socio-demographically patterned. This suggests that to ensure equity of program delivery additional consideration for those living in more deprived areas, and those who are of Black heritage is required. We recommend that public health messaging that promotes COVID-19 vaccination should focus on the regret of not being vaccinated, the benefits of a mass-immunization program, and the safety of those approved COVID-19 vaccines, and be delivered through the NHS as a trusted organization.

## Supplementary Material

Supplemental Material

## Data Availability

The data presented in this study are available upon reasonable request from the corresponding author.
