# Supplementary material for: Factors associated with vaccine intention in adults living in England who either did not want or had not yet decided to be vaccinated against COVID-19
Source: Hum Vaccin Immunother. 2021 Dec 17;17(12):5242–54. doi: 10.1080/21645515.2021.2002084 (PMC8903974; doi:10.1080/21645515.2021.2002084)
Supplement: Supplemental Material [file KHVI_A_2002084_SM7932.docx]

Table S1.

| **Coefficients** | **Standardised coefficient estimates** | **Coefficient estimates** | **Std. Error** | **t value** | **Pr(>\|t\|)** |
| --- | --- | --- | --- | --- | --- |
| (Intercept) | 0.000 | 2.879 | 0.102 | 28.323 | 0.000 |
| Age | 0.044 | 0.003 | 0.002 | 1.757 | 0.079 |
| Gender: Female | -0.015 | -0.033 | 0.054 | -0.615 | 0.539 |
| Ethnicity: Black and mixed Black | -0.053 | -0.223 | 0.106 | -2.111 | 0.035 |
| IMD | 0.067 | 0.025 | 0.009 | 2.698 | 0.007 |
| Is a health & social care key worker | -0.020 | -0.086 | 0.106 | -0.815 | 0.415 |

Adjusted R^2^: 0.01

Table S2.

| **Coefficients** | **Standardised coefficient estimates** | **Coefficient estimates** | **Std. Error** | **t value** | **Pr(>\|t\|)** |
| --- | --- | --- | --- | --- | --- |
| (Intercept) | 0.000 | 2.717 | 0.239 | 11.378 | 0.000 |
| Age | -0.035 | -0.002 | 0.002 | -1.251 | 0.211 |
| Gender: Female | -0.040 | -0.085 | 0.056 | -1.512 | 0.131 |
| Ethnicity: Black and mixed Black | -0.018 | -0.080 | 0.117 | -0.685 | 0.494 |
| IMD | 0.072 | 0.026 | 0.010 | 2.740 | 0.006 |
| Is a health & social care key worker | -0.044 | -0.191 | 0.112 | -1.707 | 0.088 |
| BMI | 0.043 | 0.008 | 0.005 | 1.602 | 0.109 |
| Previously had COVID-19 | -0.089 | -0.249 | 0.072 | -3.459 | 0.001 |
| Have been shielding from COVID-19 | -0.019 | -0.053 | 0.075 | -0.708 | 0.479 |
| General health | -0.025 | -0.033 | 0.037 | -0.905 | 0.366 |
| Flu vaccine history: increasing regularity | 0.258 | 0.175 | 0.019 | 9.338 | 0.000 |

Adjusted R^2^: 0.08

Additional variance explained by personal health factors: 0.07

Table S3.

| **Coefficients** | **Standardised coefficient estimates** | **Coefficient estimates** | **Std. Error** | **t value** | **Pr(>\|t\|)** |
| --- | --- | --- | --- | --- | --- |
| (Intercept) | 0.000 | 0.923 | 0.219 | 4.209 | 0.000 |
| Age | -0.025 | -0.002 | 0.001 | -1.108 | 0.268 |
| Gender: Female | -0.024 | -0.052 | 0.044 | -1.179 | 0.239 |
| Ethnicity: Black and mixed Black | -0.007 | -0.032 | 0.091 | -0.353 | 0.724 |
| IMD | 0.028 | 0.010 | 0.008 | 1.372 | 0.170 |
| Is a health & social care key worker | -0.031 | -0.132 | 0.087 | -1.514 | 0.130 |
| BMI | 0.026 | 0.005 | 0.004 | 1.249 | 0.212 |
| Previously had COVID-19 | -0.027 | -0.075 | 0.056 | -1.333 | 0.183 |
| Have been shielding from COVID-19 | -0.030 | -0.084 | 0.059 | -1.421 | 0.156 |
| General health | -0.012 | -0.016 | 0.031 | -0.508 | 0.611 |
| Flu vaccine history: increasing regularity | 0.132 | 0.090 | 0.015 | 5.859 | 0.000 |
| Anti-lockdown proponent | -0.168 | -0.182 | 0.024 | -7.538 | 0.000 |
| Conspiracy theorist | -0.109 | -0.120 | 0.024 | -4.971 | 0.000 |
| Perceived severity of a COVID-19 infection | 0.074 | 0.077 | 0.029 | 2.659 | 0.008 |
| Perceived susceptibility to COVID-19 infection | 0.003 | 0.003 | 0.025 | 0.105 | 0.916 |
| Trust in the NHS and the UK Government body approving a COVID-19 vaccine | 0.500 | 0.555 | 0.025 | 22.128 | 0.000 |

Adjusted R^2^: 0.45

Additional variance explained by COVID-19 pandemic belief factors: 0.37

Table S4.

| **Coefficients** | **Standardised coefficient estimates** | **Coefficient estimates** | **Std. Error** | **t value** | **Pr(>\|t\|)** |
| --- | --- | --- | --- | --- | --- |
| (Intercept) | 0.000 | -0.432 | 0.205 | -2.112 | 0.035 |
| Age | 0.004 | 0.000 | 0.001 | 0.180 | 0.857 |
| Gender: Female | -0.018 | -0.039 | 0.038 | -1.033 | 0.302 |
| Ethnicity: Black and mixed Black | 0.020 | 0.089 | 0.079 | 1.132 | 0.258 |
| IMD | 0.032 | 0.012 | 0.006 | 1.835 | 0.067 |
| Is a health & social care key worker | -0.036 | -0.155 | 0.075 | -2.068 | 0.039 |
| BMI | 0.004 | 0.001 | 0.003 | 0.230 | 0.818 |
| Previously had COVID-19 | -0.002 | -0.007 | 0.049 | -0.135 | 0.893 |
| Have been shielding from COVID-19 | -0.007 | -0.019 | 0.051 | -0.371 | 0.711 |
| General health | -0.028 | -0.037 | 0.026 | -1.387 | 0.166 |
| Flu vaccine history: increasing regularity | 0.043 | 0.029 | 0.014 | 2.140 | 0.033 |
| Anti-lockdown proponent | -0.051 | -0.055 | 0.022 | -2.465 | 0.014 |
| Conspiracy theorist | -0.001 | -0.001 | 0.023 | -0.025 | 0.980 |
| Perceived severity of a COVID-19 infection | 0.007 | 0.008 | 0.025 | 0.299 | 0.765 |
| Perceived susceptibility to COVID-19 infection | -0.027 | -0.026 | 0.021 | -1.231 | 0.218 |
| Trust in the NHS and the UK Government body approving a COVID-19 vaccine | 0.205 | 0.228 | 0.028 | 8.099 | 0.000 |
| COVID-19 vaccine attitude | 0.227 | 0.226 | 0.026 | 8.775 | 0.000 |
| COVID-19 vaccine subjective norms | 0.193 | 0.232 | 0.025 | 9.364 | 0.000 |
| COVID-19 vaccine perceived control | 0.010 | 0.010 | 0.018 | 0.521 | 0.603 |
| COVID-19 vaccine anticipated regret | 0.170 | 0.142 | 0.021 | 6.782 | 0.000 |
| Perceived safety knowledge sufficiency | 0.050 | 0.048 | 0.017 | 2.771 | 0.006 |
| Perceived benefits of COVID-19 immunisation | 0.149 | 0.214 | 0.034 | 6.308 | 0.000 |

Adjusted R^2^: 0.60

Additional variance explained by potential COVID-19 vaccine belief factors: 0.14
